# Supplementary material for: Enhanced automated detection of outbreaks of a rare antimicrobial-resistant bacterial species
Source: PLoS One. 2024 Oct 24;19(10):e0312477. doi: 10.1371/journal.pone.0312477 (PMC11500894; doi:10.1371/journal.pone.0312477)
Supplement: S1 Table — (DOCX) [file pone.0312477.s001.docx]

**S1 Table.** **Number of patients with VRE, number of beds and information source of VRE outbreak of selected hospitals with 10 and more patients with VRE between 2018-2021 in Japan.**

| Hospital | Number of patients with VRE | Number of beds | Information source of official VRE outbreaks |
| --- | --- | --- | --- |
| A-1 | 108 | 450 | WS, NP, RP |
| B-1 | 97 | 723 | N/A |
| C-1 | 88 | 388 | WS |
| D-1 | 85 | 450 | WS |
| D-2 | 72 | 577 | N/A |
| E-1 | 61 | 307 | N/A |
| B-2 | 59 | 800 | WS |
| F-1 | 53 | 500 | N/A |
| A-2 | 52 | 350 | WS |
| C-2 | 44 | 750 | WS |
| D-3 | 44 | 615 | RP |
| A-3 | 39 | 320 | WS |
| G-1 | 35 | 275 | N/A |
| D-4 | 33 | 521 | WS |
| H-1 | 32 | 470 | N/A |
| B-3 | 31 | 200 | N/A |
| D-5 | 28 | 387 | WS |
| D-6 | 23 | 520 | WS |
| E-2 | 21 | 785 | N/A |
| H-2 | 20 | 345 | N/A |
| C-3 | 20 | 400 | WS |
| I-1 | 20 | 673 | WS |
| C-4 | 19 | 218 | N/A |
| C-5 | 18 | 477 | N/A |
| C-6 | 16 | 400 | N/A |
| C-7 | 16 | 415 | N/A |
| J-1 | 15 | 1,051 | N/A |
| F-2 | 14 | 269 | N/A |
| K-1 | 14 | 800 | WS |
| B-4 | 13 | 303 | N/A |
| A-4 | 13 | 518 | WS |
| A-5 | 12 | 150 | N/A |
| C-8 | 12 | 933 | N/A |
| C-9 | 12 | 1,039 | N/A |
| C-10 | 12 | 500 | N/A |
| C-11 | 11 | 341 | N/A |
| L-1 | 10 | 702 | N/A |
| K-2 | 10 | 923 | N/A |

| Footnote | Abbreviations of "Information source of official VRE outbreaks" | |
| --- | --- | --- |
|  | WS; Web site of each hospital that can be publicly accessible | |
|  | NP; Newspaper article that reported VRE outbreaks | |
|  | RP; Research paper that targeted VRE outbreaks | |
|  | N/A; No accessible data |  |

The gray hatched hospitals are selected hospitals for cluster detection analysis in this study.
