# Supplementary material for: Enhanced automated detection of outbreaks of a rare antimicrobial-resistant bacterial species
Source: PLoS One. 2024 Oct 24;19(10):e0312477. doi: 10.1371/journal.pone.0312477 (PMC11500894; doi:10.1371/journal.pone.0312477)

**S2 Table**. Detailed information about six resistance profiles identified across 15 hospitals

| hospital | Resistance  profile  (RP) number | Antimicrobial susceptibility test result | | | | | *Number of patients with each resistance profile after de-duplication (%) | Number of statistical clusters | |
| --- | --- | --- | --- | --- | --- | --- | --- | --- | --- |
|  |  | VAN | TEC | LVX | MIN | ERY |  | space-time permutation | space-time uniform |
| A-1 | RP-1 | R | R | R | S | R | 76  (70.4%) | 0 | 4 |
|  | RP-2 | R | R | R | R | R | 23  (21.3%) | 1 | 1 |
|  | RP-3 | R | S | R | S | R | 3  (2.8%) | 0 | 0 |
|  | RP-4 | R | S | R | R | R | 5  (4.6%) | 1 | 1 |
|  | RP-5 | R | R | R | R | S | 1  (0.9%) | 0 | 0 |
|  | RP-6 | R | R | R | S | S | 1  (0.9%) | 0 | 0 |
| C-1 | RP-1 | R | R | R | S | R | 71  (80.7%) | 0 | 2 |
|  | RP-2 | R | R | R | R | R | 5  (5.7%) | 0 | 0 |
|  | RP-3 | R | S | R | S | R | 12  (13.6%) | 1 | 1 |
|  | RP-4 | R | S | R | R | R | 1  (1.1%) | 0 | 0 |
| D-1 | RP-1 | R | R | R | S | R | 67  (78.8%) | 0 | 3 |
|  | RP-2 | R | R | R | R | R | 12  (14.1%) | 1 | 1 |
|  | RP-3 | R | S | R | S | R | 8  (9.4%) | 1 | 1 |
|  | RP-4 | R | S | R | R | R | 2  (2.4%) | 0 | 1 |
| B-2 | RP-1 | R | R | R | S | R | 52  (88.1%) | 0 | 2 |
|  | RP-2 | R | R | R | R | R | 2  (3.4%) | 0 | 0 |
|  | RP-4 | R | S | R | R | R | 2  (3.4%) | 0 | 1 |
|  | RP-6 | R | R | R | S | S | 3  (5.1%) | 0 | 0 |
| A-2 | RP-1 | R | R | R | S | R | 7  (13.5%) | 0 | 1 |
|  | RP-2 | R | R | R | R | R | 42  (80.8%) | 0 | 1 |
|  | RP-4 | R | S | R | R | R | 3  (5.8%) | 1 | 1 |
| C-2 | RP-1 | R | R | R | S | R | 7  (15.9%) | 1 | 1 |
|  | RP-2 | R | R | R | R | R | 28  (63.6%) | 0 | 2 |
|  | RP-4 | R | S | R | R | R | 7  (15.9%) | 1 | 1 |
|  | RP-6 | R | R | R | S | S | 2  (4.5%) | 0 | 0 |
| D-3 | RP-1 | R | R | R | S | R | 16  (36.4%) | 1 | 1 |
|  | RP-3 | R | S | R | S | R | 28  (63.6%) | 1 | 3 |
| A-3 | RP-1 | R | R | R | S | R | 5  (12.8%) | 0 | 1 |
|  | RP-2 | R | R | R | R | R | 25  (64.1%) | 0 | 1 |
|  | RP-3 | R | S | R | S | R | 5  (12.8%) | 1 | 1 |
|  | RP-4 | R | S | R | R | R | 6  (15.4%) | 0 | 1 |
| D-4 | RP-1 | R | R | R | S | R | 2  (6.1%) | 0 | 0 |
|  | RP-3 | R | S | R | S | R | 29  (87.9%) | 0 | 1 |
|  | RP-4 | R | S | R | R | R | 2  (6.1%) | 0 | 0 |
| D-5 | RP-1 | R | R | R | S | R | 12  (42.9%) | 0 | 1 |
|  | RP-2 | R | R | R | R | R | 2  (7.1%) | 0 | 1 |
|  | RP-3 | R | S | R | S | R | 16  (57.1%) | 0 | 1 |
|  | RP-4 | R | S | R | R | R | 2  (7.1%) | 0 | 0 |
| D-6 | RP-1 | R | R | R | S | R | 15  (65.2%) | 0 | 1 |
|  | RP-3 | R | S | R | S | R | 8  (34.8%) | 1 | 1 |
| C-3 | RP-1 | R | R | R | S | R | 17  (85.0%) | 0 | 1 |
|  | RP-2 | R | R | R | R | R | 7  (35.0%) | 0 | 1 |
|  | RP-3 | R | S | R | S | R | 1  (5.0%) | 0 | 0 |
| I-1 | RP-2 | R | R | R | R | R | 6  (30%) | 0 | 2 |
|  | RP-4 | R | S | R | R | R | 14  (70%) | 0 | 2 |
| K-1 | RP-2 | R | R | R | R | R | 5  (35.7%) | 0 | 1 |
|  | RP-3 | R | S | R | S | R | 9  (64.3%) | 1 | 1 |
| A-4 | RP-1 | R | R | R | S | R | 3  (23.1%) | 0 | 1 |
|  | RP-2 | R | R | R | R | R | 1  (7.7%) | 0 | 0 |
|  | RP-4 | R | S | R | R | R | 1  (7.7%) | 0 | 0 |
|  | RP-5 | R | R | R | R | S | 8  (61.5%) | 0 | 2 |

* Percentage of patients with each resistance profile after de-duplication in parenthesis was calculated by dividing the number of patients with each resistance profile by the total number of patients with any of resistance profiles. Several different resistance profiles can be isolated from one patient.


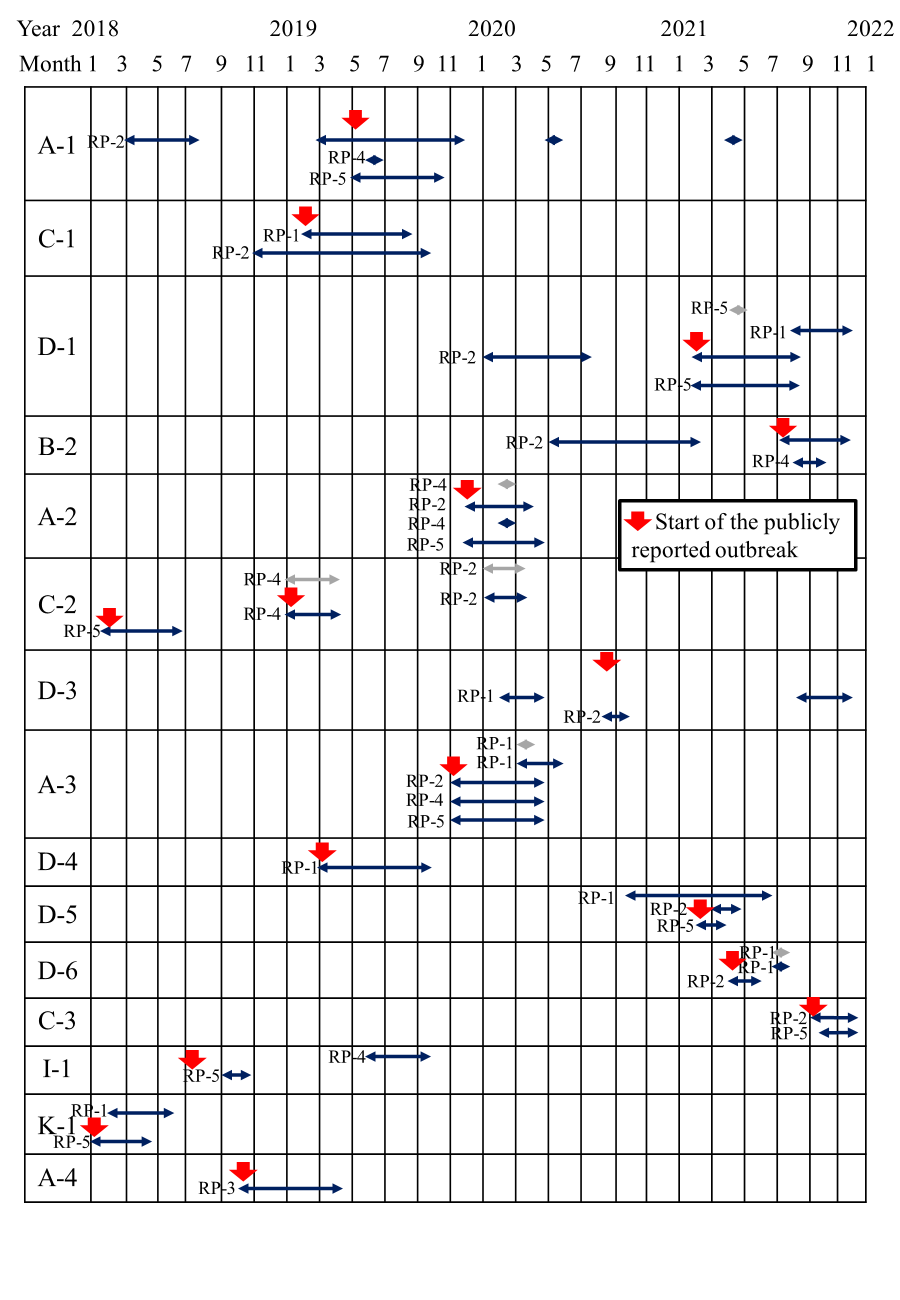

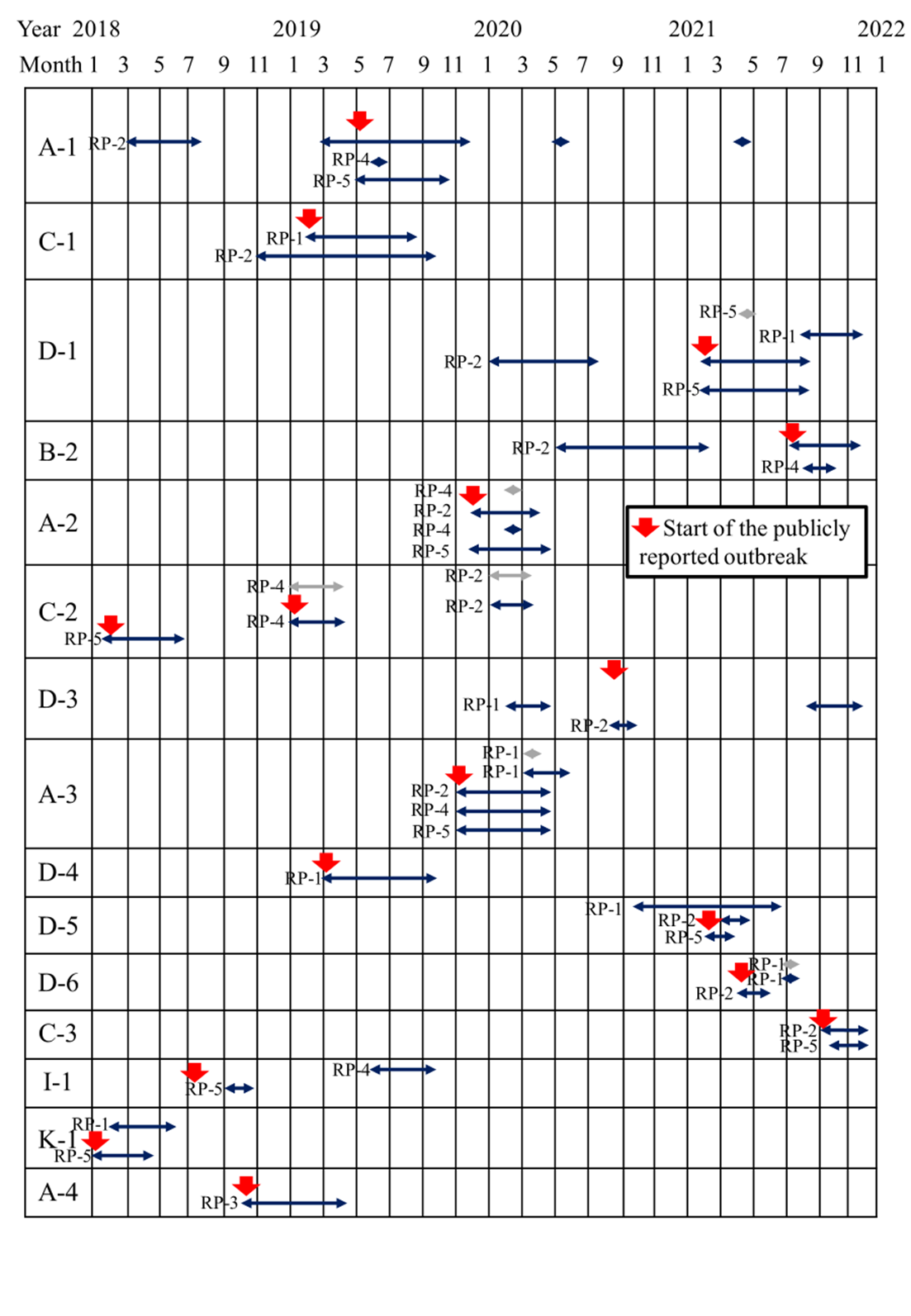

Supplement: S2 Table — (DOCX) [file pone.0312477.s002.docx]
