# Supplementary material for: Enhanced automated detection of outbreaks of a rare antimicrobial-resistant bacterial species
Source: PLoS One. 2024 Oct 24;19(10):e0312477. doi: 10.1371/journal.pone.0312477 (PMC11500894; doi:10.1371/journal.pone.0312477)
Supplement: S1 Fig — (DOCX) [file pone.0312477.s003.docx]

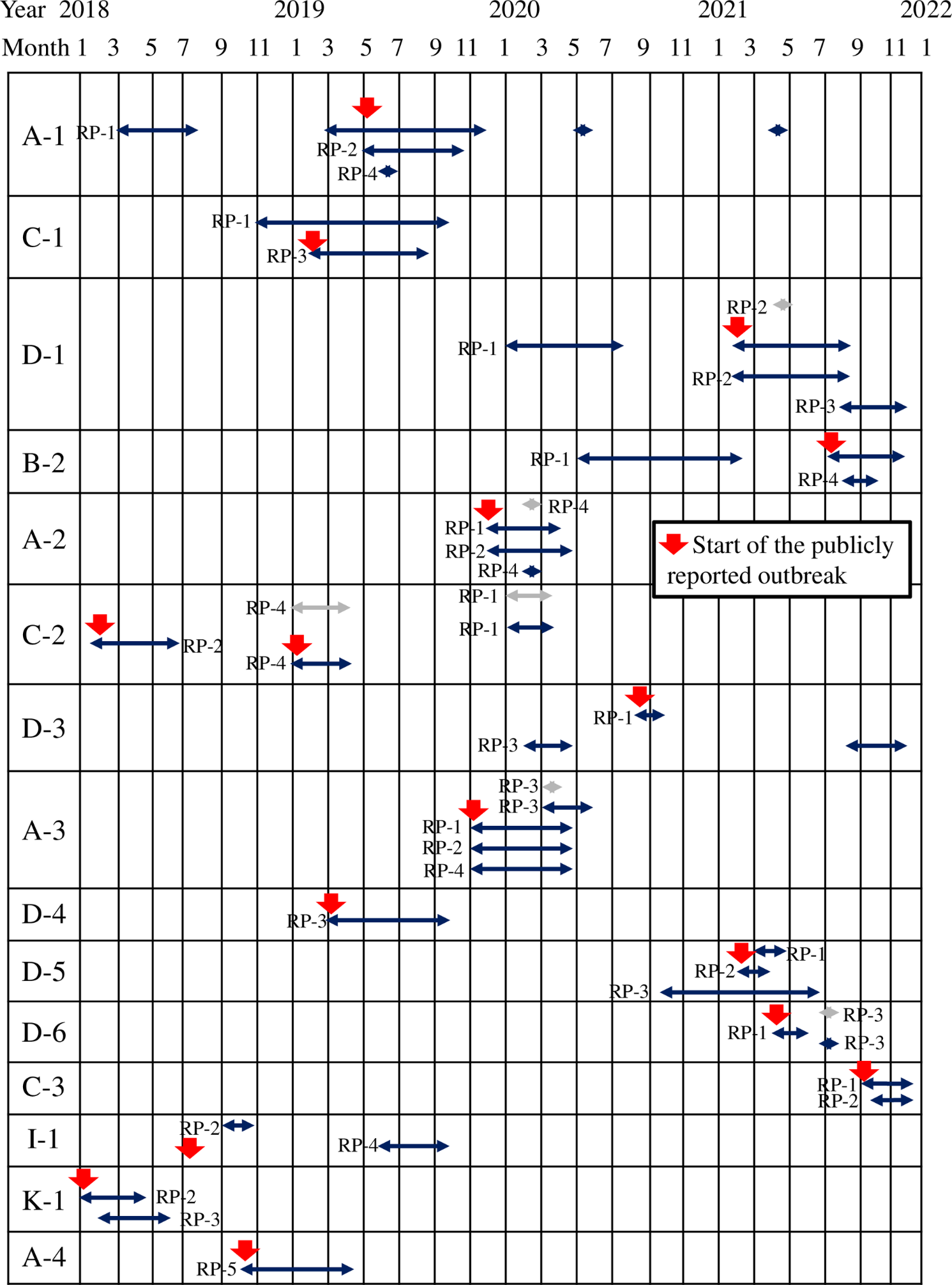
**S1 Fig.** Time course of statistical clusters detected using the space-time uniform and space-time permutation algorithms in each of the 15 hospitals and the onset of publicly reported outbreaks between January 2018 and December 2021 when the recurrence interval threshold in WHONET was changed from 30 to 365 days
