# Supplementary material for: Enhanced automated detection of outbreaks of a rare antimicrobial-resistant bacterial species
Source: PLoS One. 2024 Oct 24;19(10):e0312477. doi: 10.1371/journal.pone.0312477 (PMC11500894; doi:10.1371/journal.pone.0312477)
Supplement: Attachment — Submitted filename: response to reviewers comment.docx [file pone.0312477.s004.docx]

Reviewer #1: The manuscript describes an interesting and relevant topic regarding the use of routine antimicrobial resistance surveillance data for the automated detection of within-hospital outbreak detection.

I think the paper describes a clear research question and a clear outline of the analyses and the, and the conclusion is sound and a direct response to the proposed research question.

Still, I had a number of issues and questions while reviewing the manuscript, which I would like to mention.

My biggest concern is the fact that a relatively small number of hospitals could be included in the analyses, compared to the seemingly large surveillance system with >3,000 participating hospitals. What does that say about the generalizability of the results and the practical implications for the use of Satscan?

Response: We added the following sentences to the last paragraph in the Discussion section describing limitations of this study, based on a new flowchart (Figure 1) created according to a comment from Reviewer #2: “Fourth, only 15 hospitals with 10 or more VRE patients during the study period were included in the detailed analysis (as shown in the flowchart in Figure 1). This might be a problem in the generalization of our findings, for example among hospitals with fewer VRE patients and fewer publicly available reports on VRE outbreaks.”. The flowchart (Figure 1) which was inserted in the first paragraph of “Results” section is shown below.

And: why were those hospitals excluded if no publicly available report of a VRE outbreak was found? The lack of a regular outbreak-reporting system makes it difficult to see publicly available reports on websites as a ‘gold standard’, but perhaps the situation in Japan is suitable for this. If this is the case, then this context should be explained more clearly in the introduction / discussion.

Response: The hospitals without publicly available official reports (i.e., true positives) of VRE outbreaks were excluded in order to examine whether statistically significant clusters detected by SaTScan were associated with true outbreaks or not. We agree that it is difficult to regard publicly available reports on websites as a ‘gold standard’, but there was no other way to collect information about true positive VRE outbreaks.

Some further comments / questions:

Introduction

- Lines 48-50: ‘In hospitals, infection control teams…. Prevention and control (IPC) practices.’

In some countries, AMR surveillance and evaluation is not primarily performed by (only) infection control teams, but by medical microbiologists (usually together with ICTs). Please also mention medical microbiologists here.

Response: We added “medical microbiologists” after ICTs.

- Lines 77-79: ‘… certain hospitals have …… per hospital in recent years [8]’

The reference [8] seems not to be in place here. The sentence states that certain hospitals (plural) have reported clusters with a total number of VRE cases around 100. The paper in the reference describes one outbreak in one hospital with a total number of 60 VRE cases. Please add more references, or change the statement according to the information in the report referred to.

Response: We apologize for the inaccurate description. We changed the sentence to” there is a hospital where the total number of new VRE cases reached 60 per year, including many individuals colonized with VRE.”

Material and methods

- Line 123:

Can you please explain what methods are mostly used for phenotypically testing vancomycin-susceptibility in the laboratories? What about the possibility of false-positive results of vancomycin-resistance? Is the JANIS surveillance system only based on phenotypical antimicrobial susceptibility testing, or are genotypic testing results (e.g. vanA or vanB testing) also available? Please describe this in the Material and methods section as well.

Response: We added the following sentences to the Material and methods section: “The JANIS surveillance system is based solely on phenotypical antimicrobial susceptibility testing. The automated antimicrobial susceptibility testing methods are mostly used for phenotypically testing vancomycin susceptibility in Japanese hospital laboratories. According to recent genomic surveillance in Japan (<https://jarbs.net>), only 0.93% of the phenotypically vancomycin resistant *E. faecium* did not agree with genotypic testing results.”

- Lines 135-137: ‘To categorize each….. antimicrobial susceptibility testing’

Does the JANIS surveillance system receive all raw testing results from the participating laboratories, and could those thus be reinterpreted for these analyses according to the CLSI guidelines? Or were the laboratory interpretations (by each separate participating laboratory) used for categorizing each isolate as susceptible or resistant?

Response: Yes, the JANIS surveillance system receives all raw testing results from the participating laboratories and can thus be reinterpreted for these analyses according to the CLSI guidelines. We clarify it by modifying the sentence as “The JANIS surveillance system receives all raw testing results from the participating laboratories, and the CLSI guidelines were used to interpret the results of antimicrobial susceptibility testing and to categorize each isolate as susceptible or resistant to these antimicrobials”

- Lines 137-139: ‘Among the 96 hospitals…. the following analyses’

Why were only those hospitals included with 10 or more patients with VRE, and why not also those hospitals with 5-10 VRE during the study period?

Response: A primary reason why we focused on the hospitals with 10 or more VRE patients was to identify a sufficient number of hospitals with publicly available reports on VRE clusters for calculating sensitivity and positive predictive value of cluster detection using WHONET-SaTScan. Hospitals with a large number of VRE patients were expected to publish more reports on VRE clusters than those with a small number of VRE patients. Actually, we could not find any publicly available reports on VRE clusters in the hospitals with less than 10 VRE patients during the study period. We added “because hospitals with less than 10 patients with VRE did not have any publicly available official reports of VRE outbreaks required for validation of detected VRE clusters.” to that sentence.

- Lines 144-145: ‘showing each resistance profile of a given bacterial species per patient per surveillance period’

Only E. faecium was included, right? So it is always E. faecium as a species, or could other species be involved as well?

Response: Yes, only *E. faecium* was included, and it is always *E. faecium* as a species.

- Lines 189-195

What is common practice in Japan? Is each hospital outbreak normally reported in a public report, e.g. on the website of the hospital? Is it compulsory for hospitals to report a hospital outbreak in any publicly available report? What do you expect about underreporting? Many hospitals with relatively high numbers of VRE patients have no public report of an outbreak, does that mean that no hospital outbreak took place in those hospitals? There is one hospital with an outbreak which is reported in a research paper, but not on a website according to S1 Table. Please elaborate a bit more on clinical / routine practice in Japan.

Response: It is not the case that every hospital outbreak is normally reported in a public report. In the section of “Validation of detected VRE clusters using public reports”, we added the following paragraph: “The Infectious Disease Control Law in Japan obliges physicians to report every symptomatic case of VRE to a public health center which then sends these reports to the Ministry of Health, Labour and Welfare. Only aggregated data on the number of patients are regularly made public. However, there is no guarantee against underreporting. The absence of public reports of outbreaks in many hospitals with relatively high numbers of VRE patients does not necessarily indicate that no hospital outbreaks occurred in those hospitals. Generally, for the hospitals without publicly available VRE outbreak reports, we cannot make sure whether: 1) they had no outbreaks; 2) they had known outbreaks, but no reports are available; or 3) they had unrecognized outbreaks. “

Why were those hospitals with no publicly available official reports of VRE outbreaks during the study period excluded? I would be curious if Satscan detected clusters in any of these hospitals, and if you could find out if they concern real outbreaks, or only ‘false-positive detections’.

Response: We excluded the hospitals without publicly available official reports (i.e., true positives) of VRE outbreaks during the study period in order to calculate sensitivity and positive predictive value of cluster detection using WHONET-SaTScan. Without publicly available reports, it was impossible to classify detected clusters into true and false-positive detections. We added “in order to calculate sensitivity and positive predictive value of cluster detection using WHONET-SaTScan. Without publicly available reports, it was impossible to classify detected clusters into true and false-positive detections” to the sentence.

Results

- Line 209 / S1 Table:

The text says that 15 hospitals were chosen for the automated detection, but in S1 Table, only 13 hospitals are highlighted in grey.

Response: Thank you for pointing out our error. We have improved the S1 table and created a new version.

- Line 210: ‘after excluding 23 hospitals’

Why were these hospitals excluded? If outbreaks were publicly reported without an exact outbreak start date, you could use the date of publication as a proxy? And even if there was no publicly available information on an outbreak, why would that hospital be excluded? Perhaps no real outbreak ever took place in that hospital?

Response: We added the sentence “We could not use the publication date of the outbreak report as a proxy for the start date of the outbreak because the outbreak report is often published several weeks after the exact outbreak start date.“ after line 212. The reasons for excluding the hospitals without publicly available outbreak reports have been included in the sentence responding to your comment on lines 189-195.

- Line 215-216: ‘Satscan, which requires a comparison of multiple resistance profiles in each hospital.’

I don’t really understand this. Why does Satscan require multiple resistance profiles in each hospital? What if an outbreak happens in a hospital with only 1 particular VRE strain with isolates all having the same resistance profile? Is in that case Satscan not able to detect a cluster? Please rephrase or explain what is meant in this sentence.

Response: We apologize that the phrase was not clear enough. We modified it as “enabling statistical cluster detection for each resistance profile compared to others observed over time with SaTScan”. If an outbreak occurs in a hospital with only one particular VRE strain, where all isolates have the same resistance profile, the space-time permutation algorithm in SaTScan currently skips the cluster detection, whereas the space-time uniform algorithm conducts detection of a cluster that occurred within a specific time period.

- Line 227-229: ‘Clusters of the RP-2…. In 73.3% of the 15 hospitals.’

Why is the percentage (73.3%) of the 15 hospitals depicted in which the clusters of the RP-2 were detected? I would be more interested in the percentage of the total number of clusters (in how many hospitals) instead of the percentage of the total number of hospitals.

Response: We modified the sentence by adding the percentage of the total number of clusters, accordingly. “The number of clusters of the RP-1 mentioned above (R(VAN), R(TEC), R(LVX), S(MIN), R(ERY)) detected by the space-time uniform algorithm was 350 (50.9% of the total number of clusters) among 11 out of the 15 hospitals (73.3%).” Please note that the previous RP-2 was changed to RP-1 in this revision according to a comment from Reviewer #2.

- Table 1

Why is the total number of isolates with each resistance profile relevant? The number of isolates strongly depends on the majority of a specific outbreaks (in this the majority of all outbreaks with a specific RP), which depends on the infection prevention measures and possibility to contain the outbreak. The number of isolates for each outbreak is interesting (it shows how ‘sensitive’ the cluster detection is), but it is not really interesting to know the total number overall for each RP, I think. Perhaps you could add the range of number of patients for the detected clusters (minimum – maximum), which says more about the clusters / outbreaks.

Response: Thank you for valuable comments. We added the range of number of patients for the detected clusters (minimum – maximum) to Table 1, accordingly.

- Lines 249-252: ‘Comparing the detected statistical clusters….. for the recurrence interval.’

You don’t have a gold standard on the exact number of outbreaks, because of the possibility of underreporting, and thus, strictly, you cannot use sensitivity calculations.

Response: We incorporated your comments into the Discussion section by adding the following sentence to the last paragraph describing the limitations of this study: “Fifth, we did not have data on the exact number of true outbreaks because of the possibility of underreporting, which could bias the calculation of sensitivity.”

- Did you compare the numbers of patients with VRE within clusters detected by Satscan with the numbers of positive patients reported in the publicly available reports? Were those number (more or less) comparable?

Response: It is impossible to compare the number of patients with VRE within clusters detected by SaTScan to the numbers of positive patients reported in publicly available reports. This is because not all positive patients throughout the outbreak in each hospital are reported, and the extent of underreporting varies among hospitals.

- Lines 298-300: ‘Previously, the presence of a few….. space-time uniform algorithm.’

Do you know anything of genotyping results of these RP-2 VRE-isolates of those few patients? Could it be possible that, although they have a similar RP, that they comprise different VRE strains?

Response: We did not have any genotyping results for these RP-2 (renumbered to RP-1 in this revision) VRE isolates from those few patients.

Was this information used for feedback to the hospital, and was additional information from the hospital requested, if the outbreak might have started earlier than the publicly available starting date?

Response: The results of our study were not used to provide any feedback to the target hospitals.

Discussion

- Lines 356-357: ‘However, obtaining a precise….. of actual outbreaks.’

This also accounts for sensitivity.

Response: We modified “positive predictive value” and “sensitivity and positive predictive value” accordingly.

- Lines 363-366: ‘This suggests the possibility of an unreported true initial outbreak in 2018, followed by a subsequent true outbreak in 2019.’

See my previous question with lines 298-300: Has there been any communication with Hospital A-1 about the findings of the cluster detection? Were the ICPs possibly already aware about the positive findings in 2018 and March 2019 (without having reported publicly)?

Response: There has been no communication with Hospital A-1 about the findings of the cluster detection in this study, so it is impossible for the ICPs to be aware of the positive findings in 2018 and March 2019.

- Lines 377-383:

I agree, ward-level analyses does not always help in improved cluster detection, for example because of patients being easily transferred between different departments, or healthcare workers working on multiple departments. Could you add something about your conclusion on that? What is your recommendation?

Response: We added the following to Line 384: “Namely, the sensitivity of the ward-level analyses decreases when the number of isolates becomes small due to dividing all isolates in a hospital by ward and resistance profiles”.

- Lines 392-402:

And additionally: a similar resistance profile does not mean that multiple VRE isolates comprise one similar strain. Thus, how sure are you that all isolates within a cluster with e.g. RP-2 are from the same strain. It seems that RP-2 is a dominant resistance profile, and thus different strains will have a similar (this particular) resistance profile, and a finding of more isolates with RP-2 does not necessarily mean that there is transmission between individuals or an outbreak event.

Response: We agree, and added the following sentence to Line 403: “Relatedly, without molecular typing information, we cannot make sure whether all isolates within a cluster of a specific resistance profile are from the same strain”.

- Limitations: in the introduction and first part of Material and methods, the large coverage of the JANIS surveillance system is described as a strength, with a participation of around 8,300 hospitals. In the end, only 15 hospitals are included in the analysis for this paper, which is obviously only a very small part of the total number of participating hospitals, but still also a relatively small part of the 142 hospitals with >5 VRE during the study period 2018-2021, or of the 96 hospitals where all 5 selected antimicrobial were tested for E. faecium. Please describe this in the limitations, and perhaps add why you think this might (or not) be a problem in the generalization of your findings.

Response: We added the following to the last paragraph in the Discussion section describing limitations of this study: “Fourth, only 15 hospitals with 10 or more VRE patients during the study period were included in the detailed analysis (as shown in the flowchart in Figure 1). This might be a problem in the generalization of our findings, for example among hospitals with fewer VRE patients and fewer publicly available reports on VRE outbreaks.”

- Could this algorithm in the Satscan software also be used for multi-institutional outbreak detection? How does that work with the space-time uniform algorithm? Or is this not possible, thus how can Satscan be of practical use? And could you add that to the discussion?

Response: Yes. This algorithm in the SaTScan software can be used for multi-institutional outbreak detection through one of the following three approaches. The first approach is to look for outbreaks separately in each facility, and then check for any approximate overlap in dates or organisms across the different facilities. The second approach is to use the latitude and longitude of the facilities. The third approach is to use pre-defined relationships or groups among the hospitals instead of their latitude and longitude.

Reviewer #2: I would like to thank the authors for their work on this important aspect of infection control. Indeed, I agree that surveillance is a key part in AMR management and that automated algorithms may help IC team in this time-consuming and often tedious task.

As noted in the manuscript, VRE cases are a good candidate for the research question at hand, as they are sufficently rare for outbreaks to be noted and publicly reported. However, the fact that VRE cases are rare also has an impact of infection control practices, in the sense that strong preventive measures are usually implemented as soon a VRE positive sample is observed. Early outbreak detection is thus less critical than for other bacterial species for which strong preventive measures might require the suspicion of an epidemic cluster.

Response: Thank you. We incorporated your comments into the end of the 2^nd^ paragraph in Discussion section: “However, we should keep in mind that the rarity of VRE cases also impacts infection control practices, in the sense that strong preventive measures are usually implemented as soon as a symptomatic VRE-positive case is observed. Early outbreak detection may be more important for other bacterial species than for VRE, because strong preventive measures might require the suspicion of an epidemic cluster for the former.”

The main result of the manuscript is that the continuous uniform model of SaTScan performs better than the space-time permutation model, i.e. that it can detect more outbreaks. This is not an entirely new result (see Master’s thesis by M. Bokhari) but it has to my knowledge not been studied at this scale for hospital outbreaks. There is another algorithm, however, that sometimes shows an even higher sensitivity thhat the continuous uniform model, which is the discrete Poisson model. Can you explain why it was not mentionned or tested?

Response: The reason the Poisson model was not tested is that it requires “Population data”, meaning that the patient population in each geographic unit is needed for cluster detection based on geographical data. Since many hospitals do not have static patient population data, the Poisson-model approach is not applicable for cluster detection in this study. An advantage of the space-time uniform algorithm is that cluster detection using laboratory data is feasible without the need for external denominators, such as population data. We added these sentences to the "Detection of statistical clusters with VRE using WHONET-SaTScan" section in the Material and Methods.

One of the main issue I had reading the manuscript is that the overall description of the selection process is hard to follow: we learn line 106 that isolates from outpatients were excluded, line 137, that hospitals that don’t test for the antimicrobials chosen for the resistance profiles were excluded, as were hospitals with less than 10 VRE patients during the study period. We then learn line 182 that only data from 2018 to 2021 were used, and we finally learn lines 209-212 that 23 other hospitals were excluded because they didn’t have publicly available information about VRE outbreaks. I suggest gathering of this information in the same paragraph or section and adding a flow diagram for more clarity.

Response: Thank you for pointing out very important issue. We added a flow diagram (Figure 1, as shown below) for readers to easily understand the selection process of targeted hospitals. Accordingly, we added the following to Line 98. “The data of 1,896 hospitals were selected from the JANIS database because they had continuously provided JANIS with their microbiological data between 2018 and 2021.”

Some technical aspects of the method should in my opinion be clarified:

- Line 168; the meaning of the maximum temporal window size could be explained in more details for readers that are not well versed in this very specific statistical framework.

Response: We changed the sentence to “A maximum cluster length cut-off of 180 days was set, corresponding to the maximum temporal scanning window size for cluster generation.”

- Lines 169-170; I may have misunderstood the sentence, but how can a likelihood be expressed in days?

Response: We removed “in days” to prevent misunderstanding. The statistical likelihood that observed clusters are due to chance alone is represented by the P-value, and the recurrence interval is simply its inverse.

- The reccurrence interval threshold was set to 365 days for the sensitivity analysis; does it mean that only one outbreak per year could be expected? It seems very different from the "one outbreak per month" scenario that was used in the main analysis. Can you explain this choice theoretically or epidemiologically?

Response:　The recurrence interval threshold of 365 days does not mean that only one outbreak per year could be expected. It means that we define an outbreak as occurring under the circumstance that the observed number of patients would be expected to occur by chance less frequently than once every 365 days. Given that the threshold of 365 days has been selected to balance sensitivity with specificity in previous studies targeting AMR pathogen, including VRE [1][2][3], we selected the threshold of 365 days for the sensitivity analysis. Whether the threshold was set to 30 days or 365 days did not cause any qualitative changes in terms of sensitivity and positive predictive value for cluster detection of VRE patient in this study.

[1] Natale A, Stelling J, Meledandri M, Messenger LA, apos, Ancona F. Use of WHONET-SaTScan system for simulated real-time detection of antimicrobial resistance clusters in a hospital in Italy, 2012 to 2014. Eurosurveillance. 2017;22(11):30484.

[2] Huang SS, Yokoe DS, Stelling J, Placzek H, Kulldorff M, Kleinman K, et al. Automated detection of infectious disease outbreaks in hospitals: a retrospective cohort study. PLoS Med. 2010;7(2):e1000238.

[3] Baker MA, Yokoe DS, Stelling J, Kleinman K, Kaganov RE, Letourneau AR, et al. Automated outbreak detection of hospital-associated pathogens: Value to infection prevention programs. Infect Control Hosp Epidemiol. 2020;41(9):1016-21.

Note: these references are already cited in the manuscript.

- Baseline data for outbreak detection need to be in statistical control, meaning that there is no outbreak during this periode. Did you check for that and if so, how?

Response: There is no requirement that the baseline data needs to be in statistical control for the algorithms to detect outbreak during the study period. SaTScan can still find new outbreaks, even if there are outbreaks in the baseline period, as long as the number of new outbreak cases is large enough in a limited period of time to reach statistical significance. However, the reviewer is correct that large outbreaks of a particular organism in a particular place with a particular profile can decrease the power to find new outbreaks. To check this possibility, in this revision, we examined the number of VRE patients in each hospital during the baseline period (from 2016 to 2017) and confirmed the absence of such large outbreaks because the number of VRE patients was at most 2 in 2016 and was 4 in 2017 (only in one hospital, respectively). We added “We confirmed in advance the absence of large VRE outbreaks during the baseline period among the 15 hospitals we focus on below.” to the Materials and Methods section.

I have no particular comment regarding the antibiotics chosen for the resistance profiles, but the reasons given for their inclusion are difficult to understand at first read. I understand that TEC was chosen because it is an alternative to VAN, but why is it relevant in a context of surveillance to monitor this specific resistance? for LVX, MIN and ERY, I believe they were chosen because resistance to these antimicrobials are supposed to be more frequent, but again it is not clear as to why they are interesting for surveillance purposes. Are these resistances interesting for clinical or public health reasons? Maybe they are useful because of their discriminant value?

Response: We expected them to be useful because of their discriminant value. Namely, all five antimicrobial drugs were tested for *E. faecium* in the majority of hospitals, and we expected the inclusion of TEC, LVX, MIN, and ERY to be useful in discriminating VRE isolates in terms of AST results and resistance profiles. We added this explanation to the Materials and methods section.

The part of the manuscript that explore the results by wards in hospital D-1 is out of the scope of this study and doesn’t add anything to the research question. It should probably be a manuscript of its own.

Response:　Studies using WHONET-SaTScan (including our previous study) have mostly conducted ward-level analyses, provided the sample size per ward is not too small. We would also like to keep the results by wards in order to examine and show to what extent detected clusters change compared to the hospital-level analysis. Alternatively, we could move the results by wards to the Supplement if requested by the Reviewer and Editor.

Finally, you will find below some minor issues that I believe should be addressed:

- line 62 "A previous study ..." please be more specific. Several studies have in fact used SaTScan, even within the WHONET application, to monitor hospital outbreaks. It is important for the reader to understand from the start that the study that your are talking about is related to your team and the JANIS database.

Response: Thank you for your comment. To clarify the meaning of this sentence, we replaced “A previous study” with “Our previous study using JANIS database from 2015 to 2016” .

- line 70 "In a previous study ..." Please be more specific. Is it the same as the study mentionned line 62?

Response: Right. We changed “a previous study” to “the aforementioned study” accordingly.

- line 75 "we conducted such a comparison to detect clusters ...". I believe the sentence is misleading. The comparison was not made to detect clusters, but cluster detection performances were compared between two algorithms.

Response: We changed this sentence to “In this study, we compared the performances of two different algorithms for cluster detection of vancomycin-resistant enterococci (VRE) using the comprehensive national routine AMR surveillance data from JANIS” accordingly.

- lines 98-102: A more succint description of the JANIS hospitals would suffice, details on the distributions are in my opinion superfluous.

Response: We modified the sentence as ”Hospitals with 200 or more beds showed a higher proportion of participation in JANIS than those with fewer than 200 beds (54.4% vs. 14.6%)” accordingly.

- Lines 123-124: percentages would be more readable that fractions.

Response: We added the percentages as“number of patients with VRE/total number of patients with *E. faecium* or *E. faecalis* tested with VAN in 2022: 1,686/65,363 (2.6% of the total) for E. faecium vs 9/142,300 (0.0063% of the total) for *E. faecalis* across 2,289 hospitals participating in JANIS” accordingly.

- Line 126: VR E. faecium are already VREs, I guess what you really meant is that VREs will refer to E. faecium only.

Response: We modified the sentence as “Hereafter, VRE will refer only to vancomycin-resistant *E. faecium* in this study” accordingly.

- line 174-175: A statistically significant signal does not equal to a real outbreak indeed, but I don’t see the logical relationship of this with the start of the sentence that relate to the minimal size of the cluster.

Response: We apologize for the confusion. We removed this sentence, which we found has nothing to do with the macros used in the current manuscript.

- Lines 198-201: Although it is easily deducible, I suggest precising the country in the Ethics paragraph ("... the Japanese Ministry of Health ...", "...Japan’s Statistics Act" for more clarity.

Response: We added the country’s name to the words in the Ethics paragraph, accordingly.

- lines 206-208: these pieces of information are already stated in the methods section.

Response: These pieces of information were removed from this paragraph.

- The labelling of the resistance profiles doesn’t seem to follow any obvious scheme. Labelling them, for example, in order of their frequency might be more logical for the reader.

Response: We changed RP numbers following Table 1 where RP numbers are listed in descending order of total number and % of isolates with each RP.

- For small sample sizes, adding the count is sometimes as useful as the proportion, and I suggested adding them to the result. Line 229 for example would be "... 73.3% (n=11) of the 15 hospitals".

Response: We modified the sentence as “The number of clusters of the RP-1 mentioned above (R(VAN), R(TEC), R(LVX), S(MIN), R(ERY)) detected by the space-time uniform algorithm was 350 (50.9% of the total number of clusters) among 11 out of the 15 hospitals (73.3%)" to respond to the comments from both reviewers.

- The results should be in the past tense (e.g. line 251 "... when we useD...").

Response: Thank you for pointing out an error. We changed “use” to “used” in this sentence.

- Lines 260-261: the remark about the potential under-reporting is an element of discussion and not be in the results.

Response: We removed the remark about the potential under-reporting from Lines 260-261. The remark was already included in Discussion as “because of the potential underreporting of actual outbreaks” (Line 357).

- Line 258: I didn’t understand what "among 50 and 13 clusters detected" related to. Same remark for lines 263-264.

Response: We added “using the space-time uniform and space-time permutation algorithms, respectively” to the description.
