# Supplementary material for: Enhanced automated detection of outbreaks of a rare antimicrobial-resistant bacterial species
Source: PLoS One. 2024 Oct 24;19(10):e0312477. doi: 10.1371/journal.pone.0312477 (PMC11500894; doi:10.1371/journal.pone.0312477)
Supplement: Attachment — Submitted filename: response to reviewers comment_second revision.docx [file pone.0312477.s005.docx]

Reviewer #2: Thank you for addressing our comments, I hope you found them useful to improve your manuscript.

I only have minor issues with this new version of the manuscript, which I detail below:

I don’t understand the text added lines 77-79. Because one hospital had high rates of VRE incidence, this makes it suitable for comparison? I think you mean that VREs are rare in Japan, but that their incidence is sufficiently high to be monitored using outbreak detection algorithms. I suggest rewriting this sentence for more clarity.

Thank you for your comments. Following your suggestion, we changed the sentence to “VREs are rare in Japan, but that their incidence is sufficiently high to be monitored using outbreak detection algorithms.”

lines 123-126: this long part in parentheses containing sub parts in parentheses is are to read and contains a lot of redundancies. I again suggests rewriting this sentence for more clarity.

We appreciate your comments. We removed lots of redundancies from the long part in parenthesis.
