# Supplementary material for: Enhanced automated detection of outbreaks of a rare antimicrobial-resistant bacterial species
Source: PLoS One. 2024 Oct 24;19(10):e0312477. doi: 10.1371/journal.pone.0312477 (PMC11500894; doi:10.1371/journal.pone.0312477)
Supplement: Attachment — Submitted filename: response to reviewers comment_2.2rd revision_v2.docx [file pone.0312477.s006.docx]

Please revise your manuscript again as the two minor requests by the reviewer have not been appropriately addressed:

Line 77: Please delete "that". The correct sentence must be: VRE are rare in Japan [7], but their incidence is sufficiently high to be monitored [8] using outbreak detection algorithms.

Thank you for pointing it out. We have deleted “that” from the sentence accordingly.

Line 121: Please revise this section again. It is very hard to read.

Please report your statement in separate sentences. Please consult an English native speaker if necessary.

We appreciate your comment. We have modified the statement in separate sentences as follows: “Among several *Enterococcus* species, we focused on *E. faecium* in the subsequent analyses because this species constitutes the majority of VRE isolates in Japan and shows a higher rate of resistance to vancomycin (VAN) compared to *E. faecalis*. Specifically, rate of resistance to VAN in *E. faecium* is 2.6% (1,686 out of 65,363 isolates) for *E. faecium*, which is higher than 0.0063% (9 out of 142,300) for *E. faecalis* across 2,289 hospitals participating in JANIS in 2022)”
